# Preservation of Fertility in Cancer Patients: A Narrative Review

**DOI:** 10.7759/cureus.47910

**Published:** 2023-10-29

**Authors:** Chehak Bewtra, Neema Acharya

**Affiliations:** 1 Department of Obstetrics and Gynaecology, Jawaharlal Nehru Medical College, Datta Meghe Institute of Higher Education & Research (Deemed to be University), Wardha, IND

**Keywords:** oncofertility, male, female, cancer, fertility preservation

## Abstract

The survival rates for cancer patients have been steadily improving in recent years due to the improved efficacy of contemporary oncological care, including radiotherapy and chemotherapy. Modern technology makes it feasible to maintain fertility in cancer patients, and this practice needs to be included in oncological care. In many instances, it is impossible to avoid the harm that cancer treatments can cause to a patient's fertility; hence, research in fertility preservation techniques is being conducted to allow cancer patients to have future children biologically related to them. The development of fertility preservation approaches has grown in importance in the field of research over the past few years to increase patient's quality of life and survival. Oncologists must be aware of circumstances in which cancer patients' fertility will be impacted by their therapy and the avenues open for procedures like cryopreservation of the gametes or embryos. When cancer therapy is scheduled, all patients should receive prompt and thorough information on the fertility-related side effects of treatment and the prospects for fertility preservation. The article analyzes the literature, enlisting the factors that contribute to cancer and their effects on fertility, followed by a list of available and newly developed strategies for maintaining fertility in patients. The efficiency of the various fertility preservation techniques following cancer therapy is also discussed.

## Introduction and background

Treatment for oncological disorders may affect fertility in patients according to their age at the beginning of the treatment, its duration, severity, and type [1]. Although cancer primarily affects older people, it can also strike children, teenagers, and young adults [2]. Patients between 15 and 44 years are known to have the highest survival rates, with five-year survival rates varying from 60 to 82% depending on the age, tumor site, and country of treatment [2-4]. According to the National Cancer Registry of India, cancer incidence would rise from roughly 0.98 million in 2010 to 1.14 million annually in 2020 [5]. In essence, fertility preservation refers to preserving a person's or a couple's ability to start a family whenever they choose. The word "oncofertility" includes the maintenance of fertility in cancer patients [5]. It is increasingly crucial to take fertility preservation into account while providing these individuals with care due to improvements in the treatment of cancer linked with the genital tract like endometrial cancer and testicular cancer with increased success rates, more prolonged survival, and breakthroughs in fertility treatment. To address this, a multidisciplinary team of health experts must collaborate closely [6,7].

Impact of cancer therapy on fertility

The cornerstone methods for treating cancer are still chemotherapy and radiotherapy [5]. Given that cancer treatments have the potential to harm spermatogonia in males and ovarian follicles in female patients, cancer diagnoses in young patients who may not yet have begun families present unique concerns. If the number of spermatogonial stem cells is not entirely eliminated, spermatogenesis in males may proceed over several years [8]. Alkylating medications carry the most significant risk of long-term infertility among all gonadotoxic chemotherapy drugs [2].

Females

In young female patients, ovarian toxicity is a severe consequence of cancer treatment [9]. All female reproductive organs are susceptible to direct radiation damage if they are within the radiation field, but they can also sustain damage via scattered radiation, even in the presence of shielding [8]. It has been established that chemotherapy and irradiation are toxic to the ovaries and elevate the danger of early menopause, ovarian endocrine abnormalities, infertility, and premature ovarian failure (POF) in women [9]. Chemotherapy and radiotherapy affect the ovarian reserve to varying degrees. Chemotherapy risks vary based on the patient's age (the likelihood of ovarian failure is reduced in younger patients), the chemotherapeutic drug employed (alkylating chemicals providing the most significant risk), and the length of the treatment [10]. In women undergoing radiotherapy, concerns about fertility and hormone production are shared because both appear to be equally impacted by the treatment [1]. Radiation is particularly damaging to oocytes [10]. Acute ovarian failure and early menopause have been linked to hypothalamic, pituitary, and pelvic radiation, with or without alkylating drugs [6]. Exposure to 20-30 Gray (Gy) of radiation or 15 Gy of total body radiation can lead to a decline in ovarian function [10]. It is highlighted that doses of less than 6 Gy for adult females, less than 10 Gy for postpubertal females, and less than 15 Gy for prepubertal females are connected with a substantial risk of infertility in the pelvis or the entire abdomen [11,12]. The gonads are particularly sensitive to radiation at the prepubertal stage; half of the immature oocytes would be destroyed by radiation exposure of less than 2 Gy, and 25-50 Gy would cause infertility in one-third of young women and nearly all women over 40 years of age [12]. Young female cancer patients may experience reduced fertility due to various causes, such as cancer treatment methods, advanced age, or reproductive disorders. However, the amount and quality of the oocytes significantly contribute to the success of fertilization and embryo development. Oocyte preservation is therefore crucial whether it is done before, during, or after malignancy [9,13].

Males

Male cancer patients may experience reduced sperm transport due to poor sperm generation or reduction in the population of spermatogonial stem cells [14]. Radiation and anti-cancer medications can be harmful to the spermatogonia. Agent sensitivity specifically rises during differentiation. However, later-stage germ cells are more resilient to cell-killing impacts. Thus, the production of sperms from later-stage germ cells continues even though spermatogonia declines immediately following cancer therapy [15]. In prepubescent male patients, prolonged azoospermia can result from a dosage of 6 Gy to the testicles; however, in mature males, the limit is dropped to 2.5 Gy [16]. Early research in the late 20th century concluded that scatter radiation affected spermatogenesis even at radiotherapeutic dosages. The cells move into a senescence stage after the radiation dose is raised over 6-8 Gy with the observation that lesser doses of radiation have an enormous impact on the function of seminiferous tubules.

**Figure 1:**
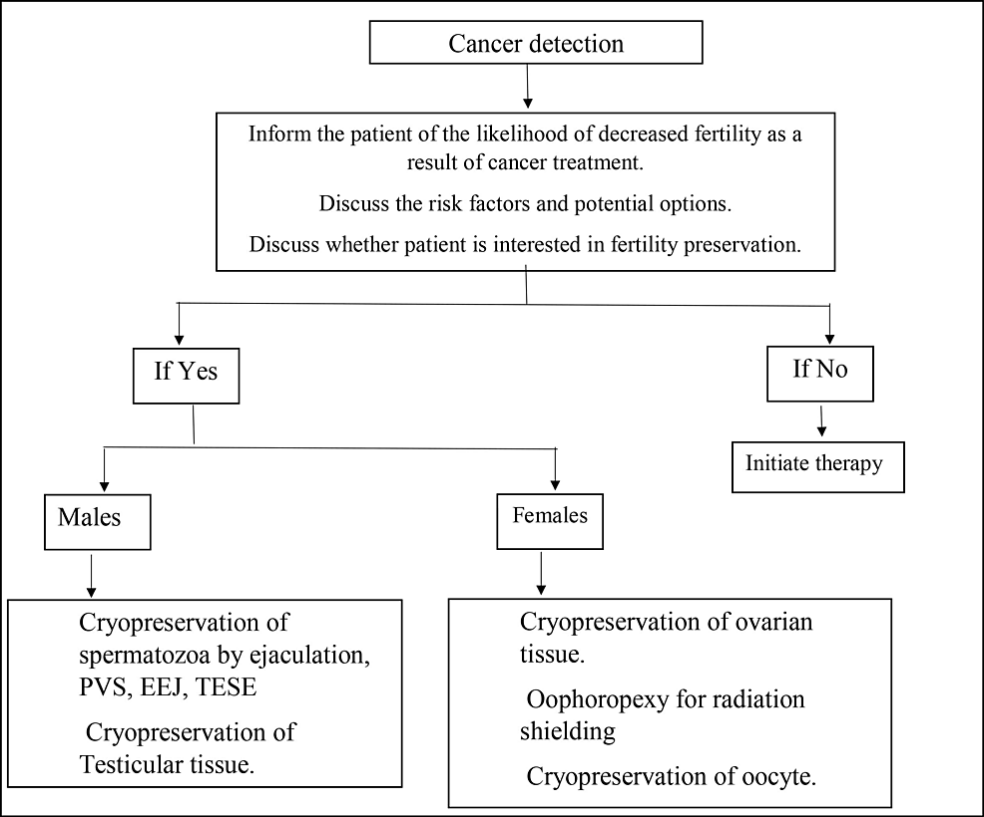
Algorithm for preservation of fertility in cancer patients PVS: Penile vibratory stimulation, EEJ: electroejaculation, TESE: testicular sperm extraction Author's own creation

Furthermore, dose fractionation appeared to impair sperm production rather than improve testicular function [15]. Leydig cells of the testis appear to be much more radiation resistant, as opposed to how it affects the generation of spermatozoa. As a result, testosterone synthesis is typically less reduced in individuals receiving even large radiotherapy [6]. Various methods of preserving fertility in both male and female patients are depicted in Figure 1. 

## Review

Methodology 

**Figure 2:**
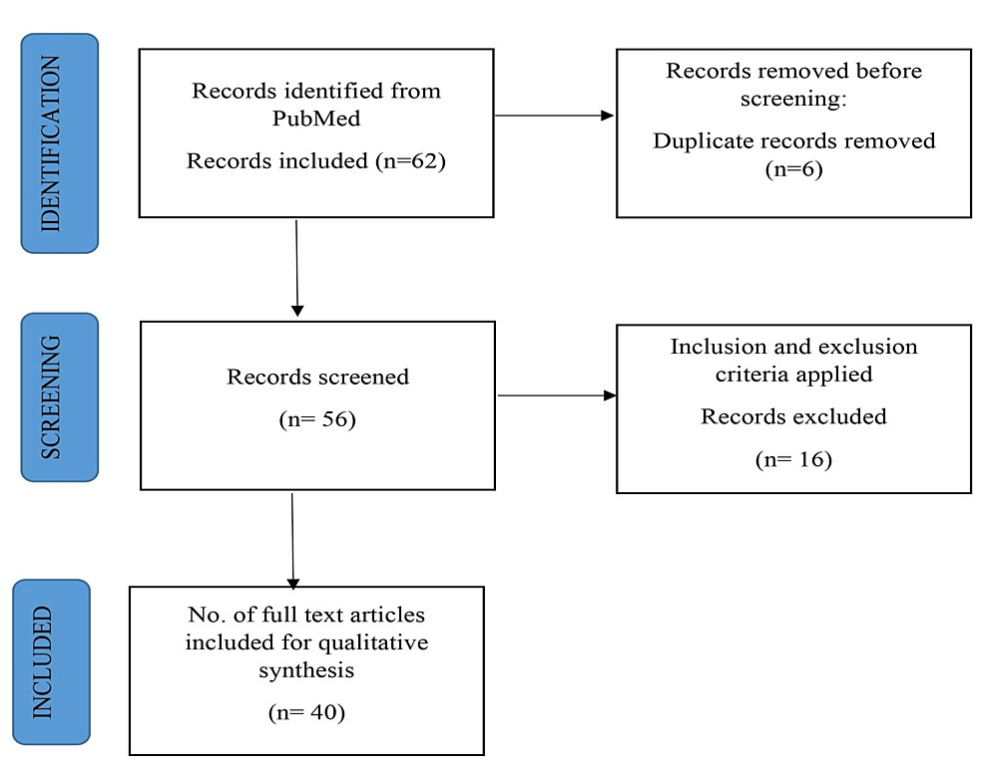
Flowchart of the methodology used for the review Author's own creation

A thorough literature search was conducted to discover relevant fertility preservation studies in cancer patients. The search was undertaken in English utilizing electronic databases such as PubMed, MEDLINE, Embase, and Google Scholar. The search terms were chosen to highlight cancer and fertility management and their impact on people at various phases of life. The subject terms chosen were “fertility preservation”, “cancer", "female", "male", and "oncofertility”. Peer-reviewed articles published in English for the secure recovery of fertility in cancer patients and the various known choices for preserving fertility in individuals undergoing cancer therapy were included in this article. Exclusion criteria included research not directly connected to fertility preservation, non-human studies, and studies that did not have full-text access. The final articles for the review were chosen after additional screening of full-text articles for potentially pertinent research. The inclusion criteria were satisfied by 40 papers included in the final review. The study selection procedure is outlined in the Preferred Reporting Items for Systematic Reviews and Meta-Analyses (PRISMA) flow diagram, shown in Figure 2. 

Fertility preservation techniques for female patients

Oocyte and Embryo Cryopreservation

For adult females, retrieval of oocytes for freezing, in vitro fertilization (IVF) of the recovered oocytes, and the subsequent cryopreservation of the fertilized oocytes are the standard procedures [8]. The woman must be married or need a sperm donor because a sperm sample is necessary to fertilize the oocyte. According to the total number and caliber of the frozen embryos, embryo cryopreservation is a well-established procedure with a high success rate [5]. The potential of embryo cryopreservation should be addressed if oncological treatment can be postponed for an oocyte stimulation cycle (particularly for patients with low and intermediate-risk Hodgkin's lymphoma and low-grade sarcomas) [6]. The main difficulties of this process include the requirement of a trustworthy male partner, legal considerations about the disposal of embryos, and the duration of ovarian stimulation [10].

Oocyte cryopreservation is a viable alternative if a sperm donor isn't available or where embryo cryopreservation isn't permitted by legislation [2,17]. The oocytes can be frozen as immature germinal vesicle oocytes or as mature eggs [10]. In situations where stimulation is impossible due to time constraints, freezing the developing eggs may also be an alternative for fertility preservation in female patients [8]. To encourage multifollicular proliferation, women receive injections of gonadotropin during controlled ovarian stimulation (COS). Egg retrieval is done after 10 to 14 days, typically with transvaginal ultrasound-guided needle aspiration and conscious anesthesia. The oocytes are cryopreserved for future use following laboratory fertilization, often at the blastocyst phase [10,18]. The various phases of the menstrual cycle are no longer a factor for these treatments [9]. According to the random start procedure by Cakmak et al. (2013), COS is initiated regardless of the phase of the cycle at the time of presentation [19]. It is crucial to understand that this is a novel procedure and that success rates are modest, possibly less than 5% per cycle [1].

Cryopreservation of Ovarian Tissue

Ovarian tissue cryopreservation (OTC) entails freezing the ovarian tissue, containing primordial follicles [9]. It involves the removal of the ovary via laparoscopic surgery, with the ovarian cortex preserved outside the body in a frozen state [20]. OTC is an appealing strategy for fertility preservation since it prevents ovarian stimulation and is the only choice for prepubertal female cancer patients [21]. The most recent Practice Committee Opinion of the American Society for Reproductive Medicine (ASRM) in January 2020 stated that ovarian tissue cryopreservation is a recognized fertility preservation treatment and is no longer deemed experimental [9,22]. The ovarian tissue can be implanted in its original site in the pelvis, i.e. orthoptic transplant, or outside the pelvis, i.e. heterotopic transplant [8]. The Lancet published a paper by Donnez et al. in 2004, which included the first successful delivery with the orthoptic introduction of the ovarian cortical tissue with a patient suffering from Stage 4 non-Hodgkin’s lymphoma, while Meirow et al. described a female who also gave birth to a second living baby and received treatment for non-Hodgkin’s lymphoma [1,23,24]. A study by Donnez et al. included a significant group of case studies in 2015, revealing 29% of the population being pregnant. Two women produced three infants each, demonstrating the technique's success and the likelihood of conceiving spontaneously following only one treatment [10,23]. While the danger of re-implanting tissue containing occult malignancy is low, it is nonetheless considerable. Further research is also being conducted to develop an "artificial ovary," in which primordial follicles are transplanted to a structural matrix, reducing the possibility of spreading cancerous cells, with success seen in mouse models [16].

Ovarian Transposition

Ovarian transposition, also known as oophoropexy, is a technique that removes the ovaries from the radiation field by detaching one or both ovaries and fallopian tubes from the uterus and securing it to the abdominal wall away from where the radiation will be directed [9]. Ovaries, however, are not always protected due to radiation dispersion, and patients should be warned that this treatment is not always successful. Because of the possibility of ovarian remigration, this procedure should be done near the radiation treatment period [25].

Other Considerations

The practice of preserving fertility has been expanding quickly, with various regimen strategies for chemotherapy-induced infertility still in the preliminary stage, awaiting clinical trial findings. Despite being less researched, various approaches have shown promise in preliminary research [8].

Cancer patients may benefit from xenotransplantation because it does not involve autotransplantation, which carries the risk of reintroducing cancer cells [26]. Nonetheless, future clinical uses of human ovarian tissue xenotransplantation are unlikely due to social and security concerns. There is cause for concern given the likelihood of zoonotic diseases, such as the transmission of prions and retroviruses from the recipient to the ovarian tissue [26,27].

Ovarian tissue cryopreserved from prepubescent patients and POF patients have immature primordial follicles that must be stimulated to grow, which can be induced in vivo by disrupting the Hippo signaling pathway or in vitro before autotransplantation [10,28].

Counseling of individuals who have breast cancer regarding the choices for preservation of fertility should go beyond the mere discussion of the effects of chemotherapy on the ovarian reserve count. The patient's age, required number of offspring, queries regarding the kind of tumor, the existing BRCA mutation, sensitivity to hormones, and concerns regarding the chance of becoming pregnant following breast cancer therapy can all differ significantly between patients [8,29]. Patients with breast cancer can have IVF while awaiting chemotherapy following surgery. The problem here is COS-induced hyperestrogenemia. To avoid high estradiol levels, aromatase inhibitors and moderate stimulation methods have been used [5].

The long-held notion that a dormant group of primordial follicles limited a woman's reproducing capacity was questioned further [30]. The ability of the hypothesized germline cells to mature in vivo has been validated in multiple studies, and it supports functionality in adult mammalian ovaries via development into cells that resemble oocytes and, ultimately, mature oocytes [31]. Stem cells have also been employed in in vitro maturation (IVM), which further involved the inclusion of human mesenchymal stem cells from both umbilical cord and menstrual blood to follicle culture and boosted follicular development, decreased programmed cell death, and enhanced survival rates [31,32]. Although human clinical studies are necessary before implementation, novel procedures paired with existing technologies to preserve fertility indicate a promising future in treatment strategies, particularly for prepubertal girls and women in urgent demand for therapy [31].

Fertility preservation techniques for male patients 

Sperm Cryopreservation

Sperm cryopreservation remains the clear alternative for males who can produce a sperm sample. It is mainly achieved by ejaculation of the semen. Additionally, it can be accomplished through testicular biopsy, testicular sperm extraction, and epididymal sperm aspiration, which can be done percutaneously or microsurgically [6,10]. In case of retrograde ejaculation, alpha-agonists or sperm extraction using urine after alkalization are recommended [11,33]. For male patients interested in retaining fertility, cryopreservation of a minimum of three sperm samples is required, with the recommendation to wait for a minimum of 48 hours between the samples [8]. Clinical pregnancy rates with thawed sperm obtained before cancer treatment range from 18% to 57% [34,35]. Although a considerable variance in the size of the testis and the existence of secondary sexual features can still exist at its commencement, the developmental stage of pubescence is regarded as a stronger predictor of spermarche. Generally, cryopreservation of sperm is provided to adolescents who have reached Tanner stage 3 of pubertal development [16,36].

Cryopreservation of Testicular Tissue 

Testicular tissue cryopreservation is still in its early stages but appears promising for fertility preservation in prepubertal children and adolescents [16]. Spermatogonial diploid stem cells may develop into mature cells after transplantation, but this procedure is still experimental. There is also a risk that testicular tissue autotransplant will reseed the cancer [2,37]. Patients should only be offered experimental techniques for fertility preservation at specialized centers under ethically authorized research methodologies and only under circumstances where the known risks associated with the operation are minor [8].

Current gaps in the information regarding preservation of fertility 

**Figure 3:**
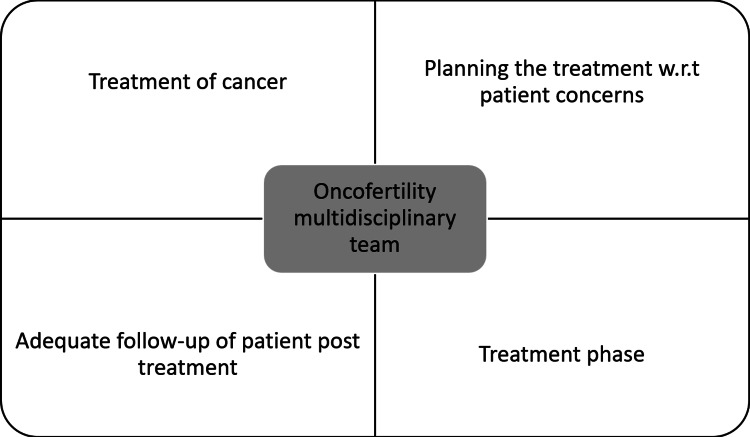
Image exhibiting the intimate link of the multidisciplinary team with the treatment algorithm of the cancer patient w.r.t - with respect to Author's own creation

Although research and studies have shown that patients who have battled cancer are prone to non-specific symptoms like stress, anxiety, and a poor quality of life, several cancer patients in their reproductive years fail to obtain enough information about fertility preservation or a recommendation to consult a reproductive specialist [8,38]. Psychosocial providers can be beneficial when a patient is worried about infertility [25]. Knowledge and attitudes of doctors towards fertility preservation have a substantial impact on the management [34,39]. A recent study found that a basic fertility training program can significantly improve oncologist's understanding of infertility risk assessment and fertility preservation techniques [34,40]. The capacity to counsel patients on the impact of specific cancer treatments on fertility is critical. It necessitates a multidisciplinary team of cancer specialists, surgeons, nurses, and fertility specialists, expanding the choices for fertility preservation for cancer patients [11]. Since the participation of experts from various sectors is critical in assuring the standard of care, we believe all such instances must be discussed in the form of a multidisciplinary team of people dealing with oncofertility, as shown in Figure 3 [11].

## Conclusions

Nowadays, many men and women are experiencing disease-free conditions in oncological healthcare. With improved chances of recovery and sustained survival, there is a need to address various options to preserve the fertility of the patients at the time of diagnosis and survivorship. The best procedure should be chosen among those available depending on the patient's characteristics: male, female, prepubertal, or postpubertal. Cryopreservation of sperm and embryos is considered standard practice and is commonly done. Other existing techniques should be treated as experimental and carried out in facilities with the required knowledge. The article focuses on addressing fertility preservation techniques for patients battling cancer and providing them with help and guidance to deal with their fertility issues. Future studies should focus on the methods to help people and healthcare professionals make decisions and prompt referrals to improve the success of fertility preservation.
